# Current perspectives on the management of patients with advanced *RET*-driven thyroid cancer in Europe

**DOI:** 10.3389/fonc.2023.1141314

**Published:** 2023-05-03

**Authors:** Rossella Elisei, Enrique Grande, Michael C. Kreissl, Sophie Leboulleux, Tarun Puri, Nicolas Fasnacht, Jaume Capdevila

**Affiliations:** ^1^ Endocrine Unit, Department of Clinical and Experimental Medicine, University of Pisa, Pisa, Italy; ^2^ Medical Oncology Department, MD Anderson Cancer Center Madrid, Madrid, Spain; ^3^ Division of Nuclear Medicine, Department of Radiology and Nuclear Medicine, University Hospital of Magdeburg, Magdeburg, Germany; ^4^ Department of Endocrinology, Diabetes, Nutrition and Therapeutic Patient Education, Geneva University Hospitals, Geneva, Switzerland; ^5^ Medical Affairs, Eli Lilly and Company, Indianapolis, IN, United States; ^6^ Medical Oncology Department, Vall d’Hebron University Hospital, Vall d’Hebron Institute of Oncology (VHIO), IOBTeknon, Barcelona, Spain

**Keywords:** thyroid cancer, tyrosine kinase inhibitor, medullary thyroid cancer, papillary thyroid cancer, receptor-tyrosine kinase, RET

## Abstract

The incidence of thyroid cancer is increasing worldwide with the disease burden in Europe second only to that in Asia. In the last several decades, molecular pathways central to the pathogenesis of thyroid cancer have revealed a spectrum of targetable kinases/kinase receptors and oncogenic drivers characteristic of each histologic subtype, such as differentiated thyroid cancer, including papillary, follicular, and medullary thyroid cancer. Oncogenic alterations identified include B-Raf proto-oncogene (*BRAF*) fusions and mutations, neurotrophic tyrosine receptor kinase (*NTRK*) gene fusions, and rearranged during transfection (*RET*) receptor tyrosine kinase fusion and mutations. Multikinase inhibitors (MKIs) targeting RET in addition to multiple other kinases, such as sorafenib, lenvatinib and cabozantinib, have shown favourable activity in advanced radioiodine-refractory differentiated thyroid cancer or *RET*-altered medullary thyroid cancer; however, the clinical utility of MKI RET inhibition is limited by off-target toxicity resulting in high rates of dose reduction and drug discontinuation. Newer and selective RET inhibitors, selpercatinib and pralsetinib, have demonstrated potent efficacy and favourable toxicity profiles in clinical trials in the treatment of *RET*-driven advanced thyroid cancer and are now a therapeutic option in some clinical settings. Importantly, the optimal benefits of available specific targeted treatments for advanced *RET*-driven thyroid cancer require genetic testing. Prior to the initiation of systemic therapy, and in treatment-naïve patients, RET inhibitors may be offered as first-line therapy if a *RET* alteration is found, supported by a multidisciplinary team approach.

## 1 Introduction

The incidence of thyroid cancer has increased over the past 50 years (1, 2). In 2020 alone, thyroid cancer was the ninth leading cause of new cancers worldwide, with an estimated 586,202 cases and an almost 3-fold greater incidence in women than men (3). Globally, the incidence of thyroid cancer continues to rise (3, 4), believed to be in large part due to factors such as an increased use of diagnostic imaging, potential overdiagnosis, and environmental and patient-related risk factors (1, 2, 5, 6). By region, the burden of thyroid cancer is reported to be greatest in Asia followed by Europe, which contributed almost 15% of the global incident (N=87,162) and mortality cases (6,399) in 2020 and 16.4% of cases (325,708) to the 5-year prevalence (3).

While the prognosis for most patients with thyroid cancer is favourable following surgical resection with/without radioactive iodine (^131^I, RAI) therapy, 5-year survival rates (<10–98%) and disease progression vary markedly by histological subtypes, which differ in morphology and gene expression (7, 8). Differentiated thyroid cancer (DTC), which is often indolent, develops from epithelial follicular thyroid cells and is the most frequent subtype accounting for 85–90% of all cases; DTC includes papillary thyroid cancer (PTC), the predominant histologic variant (>85%), and follicular thyroid cancer (FTC, 5–10%) (9, 10). Although the overall survival (OS) rate at 5 years is about 98% for most patients with DTC, local recurrence (in ~20% of cases) and distant metastases (in ~10% of cases), particularly to the lungs and bone, may occur (8). The 5-year survival rate also differs between PTC and FTC and the stage at which the cancer is diagnosed; it is almost 100% in patients with PTC, 75% in those with distant disease, and about 98% in those with FTC (63% in those with distant disease) (11). Anaplastic thyroid cancer, also known as undifferentiated carcinoma, is a rare variant (1–2%) of follicular cell origin and is an extremely aggressive cancer with mortality generally seen within months of diagnosis (9); 5-year survival rates are about 7% in patients with this subtype (8, 11). Arising from calcitonin-producing parafollicular C cells of the thyroid gland, medullary thyroid carcinoma (MTC) is also a rare thyroid malignancy, representing 3–5% of all cases; nevertheless, up to 14% of thyroid cancer-related deaths are due to MTC (12), and 5-year survival rates are 89% (40% in those with distant disease) (11). At diagnosis, about half of all patients with MTC harbour lymph node metastases and 10% have distant metastatic disease (13), with 10-year survival rates ranging from 96% for patients with intrathyroidal tumours to <40% for those with distant metastases (12). While 75% of cases of MTC occur sporadically, 25% occur as part of a hereditary syndrome, multiple endocrine neoplasias type 2 A (MEN2A), MEN2B, or familial non-MEN MTC (14, 15). Irrespective of thyroid cancer subtype, the 5-year survival rate for patients with localised disease is near 100% (11).

Alterations in signalling pathways key to the regulation of normal cell function are central to the pathogenesis of thyroid cancer – the Mitogen-Activated Protein Kinase (MAPK) and the PI3K/Akt/mTOR signalling pathways – and are potential targets for treatment. Since its identification in 1993 (16), the tyrosine kinase receptor gene, rearranged during transfection (*RET)*, which is an oncogenic driver when aberrantly activated in several malignancies including non-small-cell lung cancer (NSCLC), PTC and MTC (reviewed elsewhere; 17, 18) has emerged as an attractive therapeutic target in patients with *RET-*driven thyroid carcinoma (19).

Within the last few decades, multikinase inhibitors (MKIs), which simultaneously target kinases/kinase receptors such as the platelet-derived growth factor receptor (PDGFR), vascular endothelial growth factor (VEGF) receptor (VEGFR) 1, 2 and 3, v-kit Hardy-Zuckerman 4 feline sarcoma viral oncogene (KIT), and RET, have changed the landscape of targeted therapies for several malignancies providing initial efficacy, albeit limited by off-target activity. Next-generation selective RET inhibitors (selpercatinib and pralsetinib) have therefore been developed. This review provides a perspective on the clinical benefit of such targeted therapies in advanced thyroid cancer to date, with a focus on the present clinical management of *RET*-driven advanced thyroid cancer in Europe and the landscape of prospective advancements.

## 2 Molecular testing and challenges

### 2.1 Molecular profiles in thyroid cancer

Since the identification of the oncogenic transforming role of germline *RET* mutations in hereditary MTC almost 30 years ago, the genetic landscape of thyroid cancer has been studied extensively resulting in the identification of attractive molecular targets for small-molecule kinase inhibitors, reviewed elsewhere (20). The Cancer Genome Atlas (TCGA) study confirmed that genetic alterations are present in about 95% of cases of PTC, with a predominance of nonoverlapping mutations within the MAPK [MAPK kinase (MEK)/ERK] signalling pathway, highlighting the central role of this pathway in the onset and progression of thyroid cancer (21).

Mutant B-Raf proto-oncogene (*BRAF*) and *RAS*, and *RET* fusions were found to be the disease-causing alterations in about 80% of tumours in PTC: oncogenic *BRAF* (~60% of cases), *H-RAS* and *N-RAS* (~10%) and *RET* fusions (~5%). Other disease-causing variants identified included neurotrophic tyrosine receptor kinase (*NTRK*) fusion genes (21). Thus, DTC is generally characterised by molecular profiling as *BRAF*-predominant, *RAS*-predominant or non-*BRAF*-non-*RAS*-like, with mutations in *BRAF* and *RAS* genes being common in aggressive cancers (21, 22). Additionally, FTC is associated with *RAS* and *PAX8- PPARy* fusion disease-causing variants (23), while anaplastic thyroid cancer is associated with *TERT* promoter, *BRAF*, *RAS* or *TP53* mutations, or *NTRK* and *ALK* rearrangements (7).

Notably, *RET* alterations play a role in the pathogenesis of PTC and MTC, with *RET* gene fusions that maintain the kinase domain identified as drivers of 10–20% of all PTCs, and activating somatic or germline *RET* mutations associated with the MTC subtype (17, 24, 25). Activating mutations in the *RET* proto-oncogene are central to the development of MTC in almost all patients with a hereditary form of the disease (germline, 95–98%), and in 45−50% of those with sporadic disease (somatic) (25, 26). Somatic *RET* mutations are associated with a more aggressive phenotype, with a prevalence of up to 90% in advanced MTC (19). Most (~90%) somatic *RET* mutations are the M918T point mutation, for which the degree of aggressiveness is highest and prognosis is poor (26).

Activating *RAS* family gene point mutations (mainly *H-RAS* and *K-RAS*) which are mutually exclusive of *RET* mutations and are associated with a better prognosis, result in about 28% of cases of sporadic MTC, and *BRAF Val600*Glu (also known as *V600E*) mutations are very occasionally also found in MTC (19); for a portion of cases, the oncogenic driver has not been identified (25).

### 2.2 Importance of testing

With genetic alterations characteristic to DTC, ATC and MTC largely identified, genetic testing of patients is recommended to facilitate appropriate treatment with therapies targeted to the pathogenic pathway. Thus, the major challenges ahead in the effective treatment of *RET*-driven thyroid cancer are to identify patients at high risk of poor outcomes, their specific *RET* alterations, and to provide appropriate treatment and follow up. The identification of germline *RET* mutations may facilitate early diagnosis of hereditary MTC and somatic testing may provide information on the prognosis of sporadic MTC. The diagnostic and prognostic implications of the *RET*/PTC rearrangements in PTC are less clear but may aid in deciding if a targeted therapy should be initiated (19). In cases of apparently sporadic MTC, the identification of a *RET* germline mutation is of marked clinical utility because it facilitates the identification of subjects who will develop the tumour (27). *RET* positive relatives with no clinical evidence of MTC can be monitored with surgical treatment delayed, and *RET* negative subjects and their offspring may be reassured that they do not have any risk of developing the disease.

Testing for *RET* mutations and *RET* fusion differs (27). *RET* alteration status can be determined by immunohistochemistry (IHC), RNA or DNA-based next generation sequencing (NGS), fluorescence *in situ* hybridisation (FISH) or polymerase chain reaction (PCR), suggesting the need to identify optimal techniques for detection and differentiation (28). The European Society for Medical Oncology (ESMO) Translational Research and Precision Medicine Working Group have reviewed the available approaches for the detection of *RET* gene alterations and their potential applications (29). Recommendations from this review for the implementation of routine clinical detection of *RET* fusion genes and *RET* mutations in thyroid malignancies include the use of FISH or real-time PCR in tumours where *RET* fusions or mutations are highly prevalent, and in cancers that are rarely *RET* rearranged, broad panel assays that query *RET* fusions can be used to allow screening in a histotype-agnostic manner (29). Guidance on optimal testing for *RET* fusion and mutations include that IHC should not be used due to low sensitivity of the RET antibody(s) and multigene NGS (including *RET*) is preferred, while FISH or reverse transcriptase (RT)-PCR is indicated if NGS is not available (29). If tumour tissue is inadequate for this testing, another biopsy to obtain additional tumour tissue should be initiated, and if still inadequate, liquid biopsy with NGS (including *RET*) is recommended.

The authors of the current manuscript suggest that DNA-based NGS assay is the best approach for identifying *RET* mutations (point mutations and indels) as it also works on old tissue samples. In contrast, for *RET* fusions, the best techniques are RT-PCR or RNA-based NGS. However, old tissue samples are problematic for extracting quality RNA. If not performed earlier on, testing should be considered prior to initiation of the first systemic pharmacological therapy, as this can help to optimise the sequence of systemic treatments. An exception is germline *RET* testing in patients with MTC, which should be performed concurrently with genetic counselling at the initial diagnosis. If testing cannot be performed on the primary tumour, due to poor tissue preservation or missing tissue, re-biopsy on distant metastases or on the locally growing tumour should be taken into consideration. If contraindicated or not feasible, liquid biopsy remains an option; however, the detection of fusions using this technique requires further optimisation.

## 3 Imaging

Cervical ultrasound is reservedly the best method for the detection of locoregional recurrence of thyroid cancer and to document growth as it progresses; it is easy to perform and is a sensitive method for detecting local recurrence; however, contrast-enhanced computed tomography (CT) can also be used. Regarding further staging, the procedures differ between advanced MTC and radioiodine-refractory thyroid cancer.

If metastasis in MTC is suspected, CT of the thorax and abdomen (for lung metastases and mediastinal lymph nodes) and/or magnetic resonance imaging (MRI) of the liver is useful. MRI scans can also provide detailed images of the thyroid gland, although cost and availability may limit use compared with other imaging modalities. Alternatively, positron emission tomography (PET)/CT with a suitable radiopharmaceutical can be performed, if available. Bone scintigraphy may be conducted in individual cases, but currently it has largely been replaced by the former procedures.

There is no singular optimal PET radiopharmaceutical for the detection of MTC tumour recurrence and multiple radiopharmaceuticals have been tested. Compared with fluorodeoxyglucose (FDG)-PET/CT, ^18^F-Fluoro-dihydroxyphenylalanine (^18^F-DOPA)-PET/CT has a higher patient-based sensitivity in patients with metastatic MTC (30–34). However, both modalities provide complementary findings (32, 33); FDG-PET/CT is particularly helpful when an aggressive tumour can be assumed (35). Nevertheless, if ^18^F-DOPA-PET/CT is available, this radiopharmaceutical is used as first preference, otherwise somatostatin receptor (SSTR)-PET/CT, and in more aggressive tumours, ^18^F-FDG are used. Hence, in the clinical setting, multiple tracers may need to be evaluated for the individual patient to see if one can localise a recurrence.

An impact of PET/CT on patient management and treatment planning has been shown in 44–61% of patients (31, 36); however, these data need to be confirmed in larger patient collectives, and data on the assessment of treatment response using ^18^F-DOPA-PET or -PET/CT examination are also pending (37). Notably, PET/CT with the different radiopharmaceuticals is not only used for localisation, but also facilitates characterisation of the tumour biologically (e.g. glucose utilisation as a surrogate marker for growth behaviour or progression). The combination of examinations with different tracers can thus be useful. The use of the different radiopharmaceuticals is also described in the European Association of Nuclear Medicine guideline (38).

In the presence of metastatic radioiodine-refractory DTC, contrast CT of the thorax and abdomen (for pulmonary metastases and mediastinal lymph nodes) is required. The accuracy of FDG-PET and PET-CT in detecting DTC recurrence in patients with a negative whole-body scintigraphy has been demonstrated (39). FDG-PET or -PET/CT is consistently recommended in current European and American guidelines as complementary imaging in the setting of Tg elevation and negative radioiodine scan to search for a structural correlate for biochemical recurrence (40, 41). In aggressive histologic DTC subtypes, even if iodine-positive metastases are detected, supplemental FDG-PET/CT should be performed before initiating therapy, as detection of iodine-negative metastases in addition to iodine-positive metastases may be critical for further treatment planning. FDG-PET/CT can also be helpful for restaging during systemic therapy (42), as the remaining viability can be visualised here; bone metastases, in particular, are difficult to assess by CT or MRI in this context.

## 4 Treatment of *RET*-driven advanced thyroid cancer

### 4.1 Multikinase inhibitors in *RET*-driven advanced thyroid cancer

#### 4.1.1 *RET*-driven medullary thyroid cancer

For patients with locally advanced or metastatic MTC, the prognosis is variable, and chemotherapy and radiation therapy have not demonstrated durable objective responses in this population (13, 43); improving progression-free survival (PFS) is therefore paramount. Two MKIs that target RET, in addition to other kinases to varying degrees, are approved for the systemic treatment of MTC in the US and EU (vandetanib and cabozantinib) (44–47).

Vandetanib has inhibitory activity against VEGF2, VEGF3, EGFR, RET and – to a lesser extent – VEGF1 signalling (**Table 1**). Prolonged PFS was demonstrated with vandetanib in a phase II study in patients with locally advanced or metastatic MTC (53) and substantial prolongation of PFS was reported in a phase III trial in patients with unresectable locally advanced or metastatic MTC, 39% of whom had received prior systemic therapy for MTC (ZETA; 30.5 vs 19.3 months) (**Table 1**) (43). Based on these phase II and III results, vandetanib was the first medication to receive fast-tracked/centralised approval in the US (2011) (44) and EU (2012) (45) for the treatment of symptomatic or progressive MTC in adults, adolescents and children (aged ≥5 years) with unresectable locally advanced or metastatic disease (45). In subgroup analysis of the ZETA trial, compared with *RET M918T* mutation-negative patients, those with an *M918T* mutation had a higher objective response rate (ORR) with vandetanib (43). The EU labelling for vandetanib advises that patients without any detected *RET* mutation may have a decreased benefit from vandetanib treatment and *RET* mutation testing is recommended (45). In February 2023, a restriction of indication for vandetanib was issued by the European Medicines Agency (EMA) as based on available data from the ZETA trial (43) and *RET* status analysis, the activity of vandetanib was considered insufficient in *RET* mutation-negative patients (54). The EMA recommended that for patients in whom the *RET* mutation status is not known or is negative, vandetanib should not be administered and in such patients receiving vandetanib, the treatment should be discontinued taking into account the patients’ clinical response and the best treatment available. However, the authors note that the EMA restriction was based on ORR (not the primary endpoint of the ZETA trial) and that the type of *RET* mutation was not taken into account, which leaves *RET* mutation-negative patients with no treatment options. Additionally, a single-centre study identified factors predictive of a longer duration of response to vandetanib and a better outcome in patients with locally advanced or metastatic MTC (55). The best predictors of a longer and durable response were early treatment, younger age, good ECOG performance status and symptomatic disease, but not necessarily progressive disease; that is, those with diarrhoea or local symptoms at treatment initiation (55). Notably, in a *post hoc* analysis of the ZETA trial, compared with placebo, vandetanib demonstrated significantly improved PFS in a subgroup of 184 patients with progressive and symptomatic MTC (21.4 vs 8.4 months, hazard ratio [HR] 0.43; *p*<0.0001) (56). The most common adverse events of any grade reported with vandetanib in the ZETA trial were diarrhoea, rash, nausea and hypertension, with adverse events resulting in treatment discontinuation in more vandetanib (12%) than placebo (3%) recipients; 35% of vandetanib-treated patients required dose reduction because of adverse events or QTc prolongation (43). A phase IV trial comparing the efficacy and safety of two doses of vandetanib (150 mg/day and 300 mg/day) in patients with advanced MTC (NCT01496313) has been recently published (57). The results of this study demonstrated that the 300 mg dose showed a more favorable trend vs 150 mg as initial dose. Thus, for most patients, 300 mg vandetanib is the most appropriate starting dose; dose reductions to manage AEs and lower initial doses for patients with particular comorbidities can be considered.

**Table 1:** Summary of phase III trial results of the multikinase inhibitors (MKIs) approved in Europe for the treatment of *RET*-driven locally advanced or metastatic thyroid cancer according to Response Evaluation Criteria in Solid Tumours (RECIST).

Drug trial (Ref)	Main RTK target	Trial design, dose	Patients (N), characteristics	Primary endpoint	Secondary endpoints	Common adverse events (% of patients with event)	Dose reductions and drug discontinuation rates	Comments
MTC								
Vandetanib ZETA (43)	RET, VEGFR2,EGFR	mc, r, db, phase III trial, vandetanib, starting dose 300 mg/day po vs PBO	331; locally advanced or metastatic, hereditary (10%) or sporadic (90%) MTC^a^; prior systemic therapy (40%)	PFS: 30.5b^b^ vs 19.3 mo (HR, 95% CI: 0.46, 0.31–0.69; *p*<0.001)	PFS at 6 mo: 83% vs 63% ORR: 45% vs 13% (OR, 95% CI: 5.48, 2.99–10.79; *p*<0.001) OS: HR, 95% CI: 0.89, 0.48–1.65	>30% of patients: diarrhoea (56%), rash (45%), nausea (33%), hypertension (32%) Grade 3+ (>5%): diarrhoea (11%), hypertension (9%), ECG QT prolonged (8%), fatigue (6%)	Dose reduction: vandetanib, 81/231 (35%) PBO, 3/99 (3%) Drug discontinuation: vandetanib, 28/231 (12%) PBO, 3/99 (3%)	56% of patients had *RET* mutations. PFS was prolonged with vandetanib in patients with/without prior TKI treatment and with sporadic forms of MTC, including those with any *RET* mutation and those with *RET* ^M918T^; efficacy was observed in patients with hereditary MTC and *RET* mutations
Cabozantinib EXAM (48, 49)	RET, VEGFR2, c-KIT, MET	mc, r, db, phase III trial; cabozantinib (starting dose 140 mg/day po) vs PBO	330; locally advanced or metastatic, hereditary (6%) or sporadic (86%) MTC^c^; prior treatment^d^	PFS: 11.2 vs 4.0 mo (HR, 95% CI: 0.28, 0.19–0.40; *p*<0.001)	PFS at 12 mo: 47.3% vs 7.2% ORR: 28% vs 0% (*p*<0.001) DoR: 14.6 vs NA mo OS: 26.6 vs 21.1 mo (HR, 95% CI: 0.85, 0.64–1.12; *p*=0.24)	>30% of patients: diarrhoea (63%), palmar-plantar erythrodysaesthesia (50%), decreased weight (48%), decreased appetite (46%), nausea (43%), fatigue (41%), dysgeusia (34%), hair colour changes (34%), hypertension (33%) Grade 3+ (>5%): diarrhoea (16%), palmar-plantar erythrodysaesthesia (13%), fatigue (9%), hypertension (8%), asthenia (6%)	Dose reduction: cabozantinib 169/214 (79%) PBO, 10/109 (9%) Drug discontinuation: cabozantinib, 35/214 (16%) PBO, 9/109 (8%)	48% of patients had *RET* mutations. PFS was prolonged with cabozantinib in patients with/without prior TKI treatment, bone metastases at baseline and hereditary/sporadic forms of MTC and those with *RET* mutations; in *RET* ^M918T^-positive patients, PFS (HR, 0.15; 95% CI 0.08–0.28; *p*<0.0001) and OS (44.3 vs 18.9 mo [HR, 95% CI: 0.60, 0.38–0.94; *p*=0.03) were improved with cabozantinib
RAI-refractory DTC								
Sorafenib DECISION (50)	RET, VEGFR2,VEGFR3, c-KIT, PDGFR	mc, r, db, phase III trial; sorafenib (starting dose 400 mg bid po) vs PBO	417; locally advanced or metastatic RAI-refractory DTC (papillary [57%], follicular [25%], poorly differentiated [10%]); prior systemic anticancer therapy (3%)^e^	PFS: 10.8 vs 5.8 mo (HR, 95% CI: 0.59, 0.45–0.76; *p*<0.0001)	ORR: 12.2% vs 0.5% (*p*<0.0001) DoR: 10.2 mo vs NR Median OS not reached OS: HR, 95% CI: 0.80, 0.54–1.19; *p*=0.14	>30% of patients: palmar-plantar erythrodysaesthesia (76%), diarrhoea (69%), alopecia (67%), rash/desquamation (50%), fatigue (50%), decreased weight (47%), hypertension (41%), anorexia (32%) Grade 3+ (>5%): palmar-plantar erythrodysaesthesia (20%), hypertension (10%), decreased weight (6%), diarrhoea (5%), fatigue (5%)	Dose reduction: sorafenib 133/207 (64%) PBO, 19/209 (9%) Drug discontinuation: sorafenib, 39/207 (19%) PBO, 8/209 (4%)	PFS was prolonged with sorafenib in patients with papillary or Hürthle cell DTC, with/without bone or lung metastases at baseline and cumulative RAI ≥600 mCi
Lenvatinib SELECT (51)	RET, VEGFR1, VEGFR2,VEGFR3, c-KIT, PDGFR, FGFR	mc, r, db, phase III trial; lenvatinib (starting dose 24 mg po) vs PBO	392; locally advanced or metastatic RAI-refractory DTC (papillary [51%], follicular [37%], poorly differentiated [12%]); one prior TKI (24%)	PFS: 18.3 vs 3.6 mo (HR, 95% CI: 0.21, 0.14–0.31; *p*<0.001)	PFS at 6 mo: 77.5% vs 25.4% PFS at 12 mo: 63.0% vs 10.5% ORR: 64.8% vs 1.5% (*p*<0.001) Median OS not reached OS: HR, 95% CI: 0.73, 0.50–1.07; *p*=0.10)	>30% of patients: hypertension (68%), diarrhoea (59%), fatigue or asthenia (59%), decreased appetite (50%), decreased weight (46%), nausea (41%), stomatitis (36%), palmar-plantar erythrodysaesthesia (32%), proteinuria (31%) Grade 3+ (>5%): hypertension (42%), proteinuria (10%), decreased weight (10%), fatigue or asthenia (9%), diarrhoea (8%), decreased appetite (5%)	Dose reduction: lenvatinib 177/261 (68%) PBO, 6/131 (5%) Drug discontinuation: lenvatinib, 3/261 (14%) PBO, 3/131 (2%)	PFS was prolonged with lenvatinib in patients with papillary, follicular, Hürthle cell, poorly differentiated DTC, with/without prior TKI treatment, bone or lung metastases at baseline and cumulative RAI ≥600 mCi
Cabozantinib COSMIC-311 (52)	RET, VEGFR2, c-KIT, MET	mc, r, db, phase III trial; cabozantinib, 60 mg/day po vs PBO	187; locally advanced or metastatic RAI-refractory DTC (papillary [55%], follicular [48%]); prior lenvatinib (40%) or sorafenib (40%), or both (24%)	ORR in the first 100 randomly assigned patients: 15% vs 0% (*p*=0.028) Prespecified significance level (α=0.01) not met PFS: median not reached vs 1.9 mo (HR, 96% CI: 0.22, 0.13–0.36; *p*<0.0001)		Grade 3+ (>5%): palmar–plantar erythrodysaesthesia (10%), hypertension (9%), fatigue (8%), diarrhoea (7%), hypocalcaemia (7%)	Dose reduction: cabozantinib 70/125 (56%) PBO, 3/62 (5%) Drug discontinuation: cabozantinib 6/125 (5%) PBO, 0/62 (0%)	PFS was prolonged with cabozantinib in patients with/without prior TKI treatment

a With or without prior disease progression, no patients had only elevated calcitonin levels.

b Median PFS had not been reached for the vandetanib group, the predicted median was determined by fitting a Weibull model; the study is ongoing with completion expected end-2022 (https://clinicaltrials.gov/ct2/show/results/NCT00410761).

c Prior radiographic disease progression, no patients had only elevated calcitonin levels.

d Prior: anticancer therapy, 40%; systemic therapy, 39%; two or more systemic therapies, 25%; TKI therapy, 21% (vandetanib, 10%; sorafenib, 6%; motesanib, 3%; sunitinib, 2.7%).

e Patients who had received prior targeted therapy, thalidomide or chemotherapy for thyroid cancer were to be excluded.

Bid, twice daily; CI, confidence interval; db, double-blind; DoR, duration of response (median); DTC, differentiated thyroid cancer; ECG, electrocardiogram; EGFR, epidermal growth factor receptor; FGFR, fibroblast growth factor receptor; HR, hazard ratio; KIT, v-kit Hardy-Zuckerman 4 feline sarcoma viral oncogene; mc, multicentre; MET, hepatocyte growth factor receptor; mo, months; MTC, medullary thyroid cancer; NA, not applicable; ORR, objective response rate; OS, overall survival (median); PBO, placebo; PDGFR, platelet-derived growth factor receptor; PFS, progression-free survival (median); po, oral; r, randomised; RET, rearranged during transfection; RTK, receptor tyrosine kinase; TKI, tyrosine kinase inhibitor; VEGFR, vascular endothelial growth factor receptor.

Cabozantinib was the second drug approved in the US (2012) and EU (2014) for the treatment of progressive, metastatic MTC (46, 47). Cabozantinib inhibits the c-MET, RET and VEGF2 receptors (**Table 1**). Downregulation of the c-MET pathway may prevent invasiveness and metastatic spread, and the development of tumour resistance (58). Additionally, the latter effect may result in enhanced clinical responses compared with other MKIs. Primary analysis of the double-blind, phase III Efficacy of XL184 (cabozantinib) in Advanced Medullary Thyroid Cancer (EXAM) trial demonstrated significant improvement in PFS (11.2 vs 4 months) and ORR (28% vs 0%) with cabozantinib 140 mg/day versus placebo in 330 patients with metastatic, radiographically progressive MTC, 40% of whom had received prior anticancer therapy and 21% of whom had received prior tyrosine kinase inhibitor (TKI) treatment (**Table 1**) (48). However, in long-term follow-up, there was no difference in OS between cabozantinib and placebo (49), although subgroup analyses suggested that patients with *RET* M918T–positive tumours may experience a greater treatment benefit than other patients with MTC. Adverse events (**Table 1**) resulted in more treatment discontinuations of cabozantinib (16%) than placebo (8%) and 79% of patients required a cabozantinib dose reduction (48). In a subgroup analysis of data from 31 patients in the EXAM trial, proteinuria was found to be a late-onset (mean 38 months) adverse event with cabozantinib treatment (59).

Following the approval of cabozantinib at a dose of 140 mg/day in capsules for MTC and 60 mg/day in tablets for other solid tumours, the efficacy and safety of these two dose levels and formulations were evaluated in a phase IV randomised, double-blind, non-inferiority trial (EXAMINER) in 247 patients with progressive, metastatic MTC, 51% of whom had received prior systemic anticancer therapies, including a TKI in 41% (most commonly vandetanib; 36%) (60). For the primary endpoint, noninferiority of cabozantinib 60 mg/day tablet versus 140 mg/day capsules was not shown; median PFS in the 60 mg/day and 140 mg/day cabozantinib-treated groups were 11.0 and 13.9 months, respectively (HR 1.24; 95% confidence interval [CI], 0.90–1.70; *p*=0.19), and the ORR was 33% in each group. The rate of adverse events (Grade 3/4, 63% vs 72%), dose reductions (69% vs 81%) and treatment discontinuations due to adverse events (23% vs 36%) were lower in the 60 mg/day group.

#### 4.1.2 RAI-Refractory differentiated thyroid cancer

The prognosis of patients with DTC refractory to RAI therapy is poor, with a 10-year survival rate of 10% from the time of detection of metastasis, and limited effective therapies (51). For DTC, the standard treatment is surgical resection followed by systemic therapy with RAI in most cases (8, 61).

Potential additional therapies first targeted VEGF/VEGFR, since this signalling network is associated with the aggressiveness and metastasis of thyroid cancer; however, multiple pathways of tumour growth and maintenance and oncogene mutations contribute to thyroid cancer pathogenesis, including those of *BRAF*, *NRAS*, *HRAS*, *RET/PTC*, fibroblast growth factor receptor (FGFR), and PDGFR (20, 51). Based on the involvement of these multiple pathways, three MKIs, sorafenib, lenvatinib and cabozantinib, have been investigated for the treatment of, and subsequently received approval for, RAI-refractory DTC from the U.S. Food and Drug Administration (FDA) and the EMA (8, 61). The phase III trials that led to these approvals did not investigate the correlation between *RET* rearrangement and the efficacy of the drugs.

In the randomised, double-blind, placebo-controlled phase III DECISION trial, sorafenib, an oral MKI inhibitor of VEGFR 1, 2 and 3, platelet-derived growth factor receptor (PDGFR)β, Raf-1, RET and BRAF, showed a significant 5-month improvement over placebo in median PFS (10.8 vs 5.8 months; HR 0.59; *p*<0.0001; **Table 1**) in patients with progressive RAI-refractory DTC naïve to systemic anticancer treatment (50). The most common adverse events of any grade reported with sorafenib were palmar-plantar erythrodysaesthesia, diarrhoea, alopecia, rash/desquamation, fatigue, weight loss and hypertension (**Table 1**); 18.8% of patients discontinued because of adverse events during sorafenib therapy and 66.2% and 64.3% required a sorafenib dose interruption or reduction, respectively (50).

Lenvatinib is an oral MKI of VEGFR 1–3, FGFR 1–4, PDGFRα, RET and KIT signalling pathways (**Table 1**) (51). Following favourable results in a phase II study in patients with RAI-refractory DTC (62), the phase III Study of (E7080) Lenvatinib in Differentiated Cancer of the Thyroid (SELECT) assessed PFS in 392 patients with progressive RAI-refractory DTC that had previously been treated with ≤1 prior TKI (**Table 1**) (51). Compared with placebo, lenvatinib was associated with significant improvements in PFS (18.3 vs 3.6 months; *p*<0.001) and ORR (64.8% vs 1.5%; *p*<0.001) (51). The PFS benefit seen with lenvatinib was maintained regardless of *BRAF* or *RAS* mutation status, suggesting further investigation of biomarkers to predict a benefit with lenvatinib (51). The most common adverse events of any grade reported for lenvatinib were hypertension, diarrhoea, fatigue or asthenia, decreased appetite, decreased weight and nausea; 7.7% and 4.6% of lenvatinib and placebo recipients, respectively, died as a result of adverse events, 2.3% and 0% were considered treatment related in the lenvatinib and placebo group, respectively. In some patients, lenvatinib treatment was discontinued (14.2%), interrupted (82.4%) or the dose was reduced (67.8%) (51). In a randomised study of 152 patients with RAI-refractory DTC, compared with a starting dose of 24 mg/day, lenvatinib at 18 mg/day did not demonstrate noninferiority, as assessed by the primary endpoint of ORR at week 24, and the safety profile was comparable (63). It was concluded that these results support the continued use of the approved starting dose of lenvatinib 24 mg/day with dose adjustment as necessary. Recently, in a case series of 10 patients with advanced progressive metastatic MTC, lenvatinib showed utility as (off-label) second-line therapy (64).

Cabozantinib 60 mg/day has been assessed as second-line therapy in 187 patients with RAI-refractory DTC previously treated with lenvatinib or sorafenib, and up to two previous VEGFR-targeted therapies, in the randomised, double-blind, phase III trial cabozantinib for RAI-refractory differentiated thyroid cancer (COSMIC-311) (**Table 1**) (52). Cabozantinib significantly prolonged PFS compared with placebo: median PFS not reached (96% confidence interval [CI], 5.7–not estimable) versus median PFS of 1.9 months (1.8–3.6); HR 0.22 (96% CI, 0.13–0.36; *p*<0.0001). Grade 3/4 adverse events occurred in more cabozantinib (57%) than placebo recipients (26%). The most frequent were palmar-plantar erythrodysaesthesia (10% vs 0%), hypertension (9% vs 3%) and fatigue (8% vs 0%), while serious treatment-related adverse events occurred in 16% of the cabozantinib group and 2% of the placebo group. Based on these results, cabozantinib has been approved by the European Commission as a monotherapy for adult patients with locally advanced or metastatic DTC, refractory or not eligible for RAI who have progressed during or after prior systemic therapy (65) and by the US FDA for patients aged ≥12 years with locally advanced or metastatic DTC with progression following VEGFR-targeted therapy and who are ineligible or RAI refractory (66).

**Figure 1:**
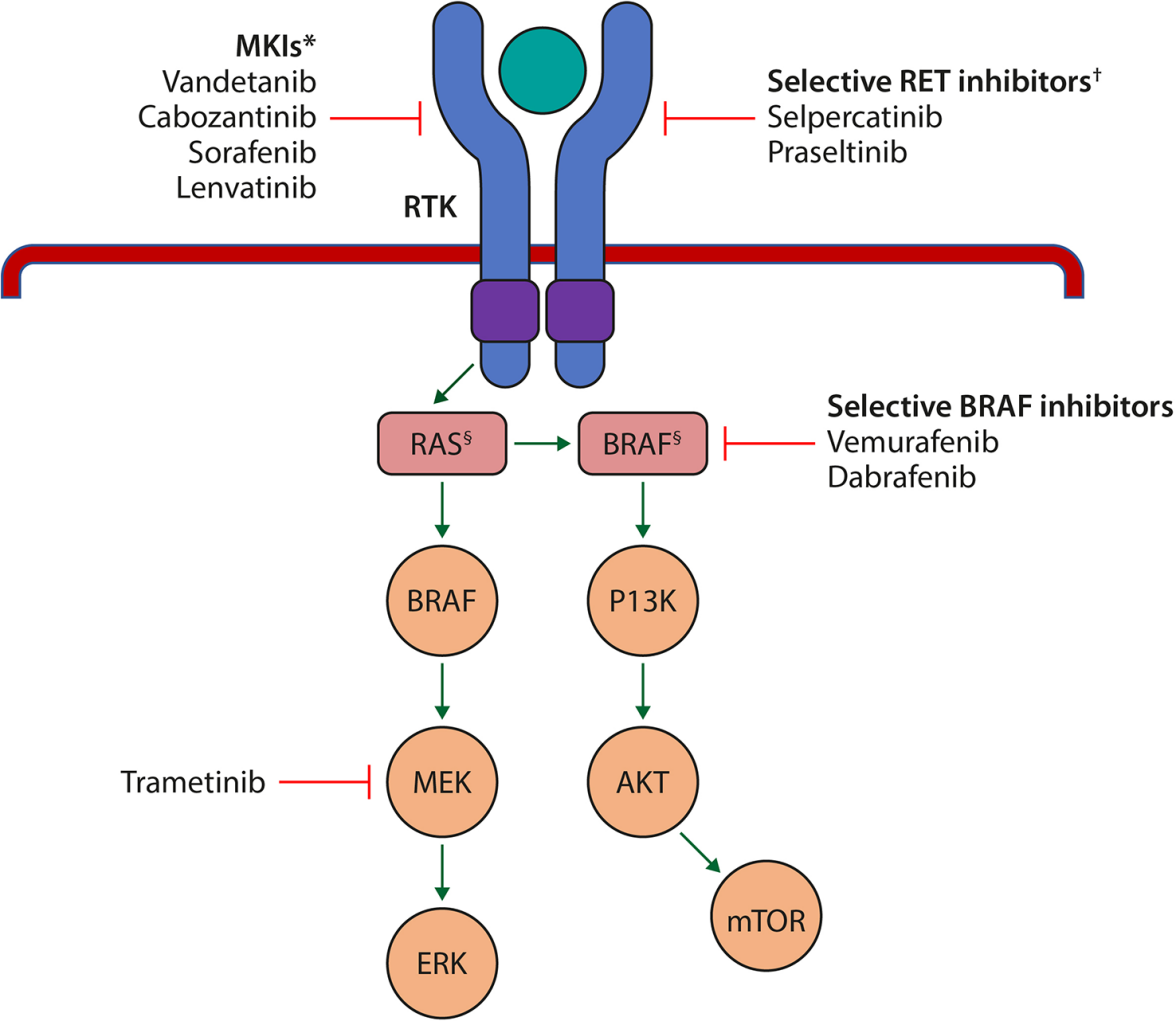
Schematic representation of the key signalling pathways in cell growth and proliferation that are affected in the pathogenesis of thyroid cancer, and the sites of action of the currently approved MKIs and selective RET inhibitors. *Target several RTKs simultaneously such as the VEGFR isoforms, FGF; c-KIT, RET, PDGFR and EGFR. †Selectively target mutant RET kinase. §Harbour mutations. AKT, protein kinase B; BRAF, B-Raf proto-oncogene, serine/threonine kinase; c-KIT, cellular mast/stem cell growth factor receptor; EGFR, epidermal growth factor receptor; ERK, extracellular signal-regulated kinase; FGF, fibroblast growth factor; MEK, mitogen activated protein kinase; MKI, multikinase inhibitor; mTOR, mammalian target of rapamycin; PI3K, phosphoinositide 3-kinase; PDGFR, platelet-derived growth factor receptor; RAS, rat sarcoma; RET, rearranged during transfection; RTK, receptor tyrosine kinase; VEGFR, vascular endothelial growth factor receptor. Adapted from Porter A and Wong DJ. *Front. Oncol.* 2021 (67).

Although the MKIs targeting RET have shown activity in MTC and RAI-refractory DTC, the efficacy of MKI RET inhibition has been limited by the frequency of adverse events and off-target toxicity associated with more potent inhibition of non-RET kinases, particularly VEGFR2, suggesting a need for improved, more specific RET-targeting therapies (**Figure 1**) (13).

### 4.2 Selective RET inhibitors in advanced thyroid cancer

Two novel selective RET inhibitors with efficacy in advanced *RET*-altered cancers have been developed, selpercatinib and pralsetinib (**Table 2**). Available data show that these agents, which have less activity against *VEGFR2* than *RET* alterations such as *M918T* and other mutations or fusions, are well-tolerated and highly active, regardless of the type of *RET* mutation. Based on the efficacy and safety of selpercatinib and pralsetinib in phase I/II studies in *RET*-altered NSCLC and/or thyroid cancer (68, 71, 73), in the both selpercatinib and pralsetinib received approval for the treatment of selected adults with locally advanced or metastatic (selpercatinib)/metastatic (pralsetinib) *RET* fusion-positive NSCLC and accelerated approval for adults and adolescents aged ≥12 years with advanced or metastatic *RET* mutation-positive MTC who require systemic therapy or with advanced or metastatic *RET* fusion-positive thyroid cancer who require systemic therapy and are radioactive iodine-refractory (77, 78). Notably, in 2022, selpercatinib was granted accelerated approval for adults with locally advanced or metastatic *RET* fusion-positive solid tumours following systemic treatment or who have no satisfactory alternative treatment options; this was the first tumour-agnostic approval for *RET* fusion-positive cancers in the US (79).

**Table 2:** Summary of phase I/II trial results of the selective RET inhibitors approved in Europe for the treatment of *RET*-driven advanced thyroid cancer according to Response Evaluation Criteria in Solid Tumors (RECIST).

Drug trial (Ref)	Target receptors	Trial design, dose	Patients (N), characteristics	Primary endpoint (95% CI)	Secondary endpoints	Common TRAEs (% of patients)	Dose reductions and drug discontinuations	Comments
Selpercatinib LIBRETTO-001 (66, 67) (follow-up to March 2020 plus 15 months)	RET	mc, ol, phase I trial of selpercatinib 20–240 mg bid po; phase 2 trial of selpercatinib 160 mg bid po	151, *RET*-mutant MTC; previously treated with vandetanib and/or cabozantinib	ORR: 73.5% (65.7–80.4)	PFS at 2 years: 64.4% (55.4–72.0) Median PFS: 34 mo (25.7–NE) after median follow-up 27.6 mo Median DoR: NE (27.2–NE)	>20% of patients with MTC (N=319): dry mouth (36%), hypertension (32%), fatigue (35%), oedema (34%), diarrhoea (25%), increased aspartate aminotransferase level (26%); increased alanine aminotransferase level (25%); constipation (21%); Grade 3+ (>5%): hypertension (14%), increased alanine aminotransferase level (7%), increased aspartate aminotransferase level (6%)	In patients with MTC: Dose adjustment: 116/319 (36%) Drug discontinuation due to TRAEs: 13/319 (4%)	Efficacy was observed regardless of the number of previous MKIs and across all qualifying *RET* mutations, including *RET* V804
LIBRETTO-001 (67) (follow-up to March 2020 plus 15 months)			142, vandetanib- and cabozantinib-naïve *RET*-mutant MTC	ORR: 81.0% (73.6–87.1)	PFS at 2 years: 81.1% (72.4–87.3) Median PFS mo: NE (NE–NE) after median follow-up 24.5 mo Median DoR mo: NE (NE–NE)			
LIBRETTO-001 (follow-up to March 2020) (68)			22, previously treated non-MTC *RET* fusion-positive TC	ORR: 77.3 (54.6–92.2)	PFS >1 year: 92.9% (84.5–96.8) DoR: 18.4 mo (10.1–NE)	Treatment-emergent adverse events: dry mouth, diarrhoea, hypertension, fatigue, constipation, nausea (≥25% for all)	Drug discontinuation due to TRAEs: 0/22 (0%)	
Pralsetinib ARROW (69, 70)	RET	mc, ol, phase I/II trial of pralsetinib 400 mg/day; phase 2 trial of pralsetinib 400 mg/day po	67, *RET*-mutant MTC previously treated with vandetanib and/or cabozantinib	ORR: 52.2% (39.7–64.6)	Median PFS: 25.8 mo (19.7–35.0) Median DoR: 25.8 mo (18.0–NE)	Serious TRAEs, 26/145 (17.9%) including pneumonitis in 2.8%; Grade 3+ (62.8%)	Dose reduction due to TRAEs: 77/145 (53%) Drug discontinuation due to TRAEs: 8/145 (6%) Death: 1/175 (<1%) (*pneumocystis jirovecii* pneumonia)	Efficacy was observed in patients with *RET*-altered locally advanced or metastatic solid tumours, including *RET*-mutant MTC, previously treated, *RET*-mutant MTC and *RET* fusion-positive thyroid cancers
			67, vandetanib- and cabozantinib-naïve *RET*-mutant MTC	ORR: 71.6% (59.3–82.0)	Median PFS: NR (27.5–NE) Median DoR: NR (NE–NE)			
			25, *RET* fusion-positive TC, previously treated with systemic agents	ORR: 84.0% (63.9–95.5%)	Median PFS: 25.4 mo (17.0–NE) Median DoR: 23.6 mo (15.1–NE)	Serious TRAEs, 3/30 (10.0%) including anaemia, dizziness, hypotension and pneumonitis; Grade 3+ (53.3%)	Dose reduction due to TRAEs: 15/30 (50%) Drug discontinuation due to TRAEs: 2/30 (7%)	

Bid, twice daily; CI, confidence interval; DoR, duration of response; mc, multicentre; MKI, multi-kinase inhibitor; MTC, medullary thyroid cancer; mo, months; NE, not estimable; NR, not reached; ol, open-label; ORR, objective response rate; PBO, placebo; PFS, progression-free survival; po, oral; RAI, radioactive iodine; RET, rearranged during transfection; RTK, receptor tyrosine kinase; TC, thyroid cancer; TKI, tyrosine kinase inhibitor; TRAE, treatment-related adverse events.

Additionally, selpercatinib has been approved as monotherapy throughout the EU in adults with advanced RET fusion-positive thyroid cancer requiring systemic therapy following prior treatment with sorafenib and/or lenvatinib (74), and has been approved as first-line monotherapy in adults and adolescents aged ≥12 years with advanced RET-mutant MTC (76). No approval for the treatment of thyroid cancer, neither DTC with RET fusions nor MTC with RET mutations, has been granted for pralsetinib so far. Pralsetinib in EU has been approved only for treating adults with advanced NSCLC caused by RET fusions and who have not been treated with a RET inhibitor.

#### 4.2.1 Selpercatinib in *RET*-altered advanced thyroid cancer

The efficacy and safety of selpercatinib, a first-in-class highly selective and potent RET kinase inhibitor, was evaluated in LIBRETTO-001, a phase I/II trial in a total of 531 patients with *RET*-altered (fusion or mutation) advanced or metastatic cancer, including three thyroid cancer cohorts (68). These cohorts were: *RET*-mutant MTC previously treated with vandetanib and/or cabozantinib (n=55; the primary analysis set [PAS]); *RET*-mutant MTC not previously treated with vandetanib and/or cabozantinib (n=88); and previously treated *RET*-fusion positive thyroid cancer (n=19). ORRs per RECIST v1.1 by independent review in the three cohorts treated over an initial 2-year period were 69%, 73% and 79%, respectively, with responses across all *RET* alterations and histologies; median duration of objective response and PFS were not reached at a median follow-up of 14.1 and 16.7 months, respectively. Selpercatinib showed durable efficacy and mostly low-grade adverse events. The most common adverse events of grade 3 or higher were hypertension (21%), increased alanine aminotransferase (11%) and aspartate aminotransferase (9%) levels, hyponatraemia (8%) and diarrhoea (6%); 2% of patients discontinued selpercatinib because of treatment-related adverse events.

In updated analyses of LIBRETTO-001 with longer follow up, selpercatinib treatment in patients with *RET*-altered thyroid cancers continued to show marked and durable antitumour activity (**Table 2**) (69, 70). In an integrated analysis set (IAS), which was an expanded population of efficacy evaluable patients with *RET*-mutant MTC previously treated with cabozantinib and/or vandetanib (N=151) and in an expanded cohort of cabozantinib and/or vandetanib naïve patients with MTC (N=142), ORRs were 73.5% (at a median follow-up of 22.9 months) and 81.0% (median follow-up, 20.3 months), respectively. In an earlier analysis, patients with previously treated non-MTC *RET* fusion-positive thyroid cancer (N=22) had an ORR of 77.3%. The proportions of patients with *RET*-mutant MTC who were alive and progression free at 2 years were 64.4% and 81.1% in the pre-treated and treatment-naïve cohorts, respectively (**Table 2**). Selpercatinib treatment continued to be well-tolerated after up to 2 years of treatment (69, 70). The most common treatment-related adverse events in the safety population who received ≥1 dose of selpercatinib (MTC, N=319; non-MTC thyroid cancer, N=42) included dry mouth, fatigue, hypertension, oedema, diarrhoea, increased aspartate/alanine aminotransferase levels and constipation, which were predominantly low grade (**Table 2**) (69, 70).

Selpercatinib is a small-molecule, highly selective inhibitor of RET kinase which can penetrate the central nervous system (CNS) and has been shown in preclinical studies to have antitumour activity in the brain (80). The CNS activity of selpercatinib has also been shown in a NSCLC cohort, with an 85% (95% CI, 65–96%) CNS ORR reported in 26 patients with CNS metastases at baseline (81). Moreover, in a patient with advanced metastatic sporadic MTC treated with selpercatinib, a significant and durable clinical response of choroidal metastases has been recently reported (82).

#### 4.2.2 Pralsetinib in *RET*-altered advanced thyroid cancer

Pralsetinib is another oral, once-daily, selective RET inhibitor. The efficacy and safety of pralsetinib were assessed in ARROW, a phase I/II trial conducted across 13 countries worldwide in patients with *RET*-altered locally advanced or metastatic solid tumours, including NSCLC, and *RET*-mutant MTC and *RET* fusion-positive thyroid cancer (71). In the most recent analysis disclosed for this study, patients who had initiated pralsetinib 400 mg/day before the enrolment cut-off were included in the intention-to-treat population, and those who had initiated pralsetinib prior to data cut-off were included in the safety population (72).

Respective ORRs in the three cohorts of patients with previously treated (vandetanib and/or cabozantinib) *RET*-mutant MTC (n=67), treatment-naïve *RET*-mutant MTC (n=67), and previously treated *RET* fusion-positive thyroid cancer (n=25) were 52.2%, 71.6% and 84.0% (72). Median duration of objective response and PFS were not reached in the treatment-naïve *RET*-mutant MTC cohort as of 18 October 2021, but were 25.8 and 25.8 months, respectively, in the previously treated *RET*-mutant MTC cohort, and 23.6 and 25.4 months in the *RET* fusion-positive thyroid cancer cohort (72).

The incidence of treatment-related adverse events among the safety population of patients with *RET*-altered thyroid cancer was 97.9% in the MTC cohort (142/145) and 93.3% in the non-MTC thyroid cancer cohort (28/30) (72) (**Table 2**). Serious treatment-related adverse events were reported in 16.6% of the 175 patients; among patients with *RET*-mutant MTC, the most frequent was pneumonitis (2.8%), and among the *RET*-fusion positive patients, one event each of anaemia, dizziness, hypotension and pneumonitis was reported. Ten patients (5.7%) discontinued treatment because of treatment-related events, 92 (52.6%) required dose reduction and there was one death associated with a treatment-related adverse event (72). Overall, in the updated analysis of the ARROW trial with longer follow up of patients with *RET*-altered thyroid cancer (72), pralsetinib continued to show efficacy along with a manageable safety profile.

## 5 Guideline recommendations for patients with thyroid cancer

Oncology treatment guidelines are regularly updated to reflect latest clinical trial results, and provide best evidence-based recommendations. For the management of patients with DTC with distant metastatic disease that is RAI refractory and for patients with MTC with metastatic disease, a period of ‘watchful waiting’ may be appropriate, as the clinical outcome may be indolent despite the presence of metastatic disease. For patients with DTC or MTC for whom localised therapies have failed, or in whom these therapies are not feasible and who have aggressive disease progression, systematic therapy is recommended (7, 61, 83). Initiating treatment earlier, when the tumour burden is smaller, may lead to more favourable outcomes. Nevertheless, the use of MKIs and newer RET-selective inhibitors in the real-world setting is influenced by the regulatory heterogeneity across countries.

The National Comprehensive Cancer Network (NCCN) guidelines (V 2.2022) for thyroid carcinoma provide specific recommendations for DTC (PTC and FTC) and MTC (83). Updates to the guidelines include the expanding role of molecular testing, with recommendations for genetic testing for *RET* mutations in patients with clinically apparent sporadic MTC, and screening of children and adults with relatives with known hereditary MTC.

According to the NCCN, systemic TKI therapy as a first-line regimen, vandetanib or cabozantinib, should be considered in unresectable locoregional diseases and distant metastases with symptomatic or progressive MTC by RECIST (83). A selective RET inhibitor, selpercatinib or pralsetinib, can be considered for patients with a positive *RET* somatic mutation; that is, *RET*-mutated MTC confirmed by germline testing including tumour mutational burden (TMB) or *RET* somatic genotyping in patients who are germline wild-type or germline unknown (83).

The FDA-approved MKIs lenvatinib or sorafenib are recommended as first-line systemic therapy for locally recurrent, advanced, and/or metastatic RAI-refractory DTC; cabozantinib may be considered in the case of progression after lenvatinib and/or sorafenib. In 2021, for the first time, NCCN guidelines recommended the selective RET inhibitors selpercatinib and pralsetinib for patients with advanced or metastatic *RET* fusion-positive DTC.

ESMO guidelines highlight that the main goals for the treatment of thyroid cancer are improving OS and quality of life. Moreover, it is recognised that to minimise the risk associated with overtreatment, more aggressive diagnostic and therapeutic options are required for higher-risk patients versus those with indolent tumours (7, 61). As such, ESMO and the European Thyroid Association (ETA) clinical practice guidelines for thyroid cancer recommend that all patients with clinically apparent sporadic MTC are offered genetic counselling and screening for germline *RET* mutations. Additionally, if treatment of advanced MTC with selective RET inhibitors is planned, testing for somatic *RET* mutations is recommended to individualise therapy (61, 84). EMA-approved MKIs are considered by ESMO as the standard first-line systemic therapy for progressive, locally advanced or metastatic DTC refractory to RAI (lenvatinib or sorafenib) or MTC (cabozantinib or vandetanib [including children aged ≥5 years]) (7, 61). Additionally, cabozantinib may be considered in adults and paediatric patients (aged ≥12 years) with locally advanced or metastatic DTC that has progressed following VEGFR-targeted therapy and who are RAI-refractory or ineligible.

Selpercatinib is recommended by ESMO for adults with advanced *RET* fusion-positive DTC requiring systemic therapy following prior treatment with sorafenib and/or lenvatinib and is also approved by the EMA for this indication. Pralsetinib treatment may be considered for adults and paediatric patients (aged ≥12 years) with advanced or metastatic *RET* fusion-positive DTC requiring systemic therapy and who are RAI-refractory (7, 61). However, in EU pralsetinib is not approved for the treatment of any type of thyroid cancer and application of its use in thyroid cancer was withdrawn from the EMA in November 2022 (85). In the MTC setting in the US, selpercatinib or pralsetinib may be considered for systemic therapy in adults and paediatric patients (aged ≥12 years) with advanced, but not pralsetinib, or metastatic *RET*-mutant disease (74, 75; in Europe, selpercatinib is approved for *RET*-mutant MTC in this population (76).

## 6 Precision-targeted RET inhibitors and precision oncology

Investigations into the efficacy and safety of selpercatinib in a *RET* fusion-positive tumour-agnostic population of 45 patients with non-lung, non-thyroid advanced solid tumours in LIBRETTO-001 showed clinically meaningful efficacy, an ORR of 43.9% (95% CI, 28.5–60.3%) in 41 efficacy-evaluable patients, and a safety profile similar to that seen in other indications (86). It was concluded that comprehensive genomic testing that includes *RET* fusions will be key in identifying candidates for targeted RET inhibition.

Similarly, the ARROW trial included 29 patients with diverse non-lung, non-thyroid advanced *RET* fusion-positive solid tumour types. Pralsetinib demonstrated rapid and durable anti-tumour activity in the 23 efficacy-evaluable patients, and was well tolerated (87). In the tumour agnostic cohort, ORR was 57% (95% CI, 35–77%) and median duration of response, PFS and overall survival were 12 months, 7 months and 14 months, respectively, validating RET as a tissue-agnostic target for RET inhibition, which is crucial for precision medicine in cancer.

The clinical success and approval of selective RET inhibitors for MTC and PTC, with fewer off-target adverse events and more effective and sustained anti-tumour activity compared with MKIs, have facilitated thyroid cancer entering precision oncology for the RET-dependent cancers, *RET*-mutant MTC and *RET* fusion-positive PTC.

Thyroid cancers are perfect for precision oncology that originates and derives from the knowledge of the oncogene profile of thyroid tumours. Overall, about 80% of thyroid cancers have oncogenic alterations; however, not all oncogenic alterations are treatable with pharmacotherapies and this presents a challenge in identifying new agents for each oncogenic alteration. Additionally, there may be disparities between European countries in the rate of DTC with an unidentified oncogenic driver.

In addition to selective RET inhibition for the treatment of patients with *RET* alterations, inhibitors of *NTRK* fusions (tropomyosin receptor kinase [TRK] inhibitors) and *BRAF* mutations (BRAF inhibitors) have either been approved or have shown promising activity in thyroid cancer (88, 89). Discussion of these targeted therapies is beyond the scope of this review.

## 7 Current challenges

The prognosis of patients with metastatic *RET*-altered thyroid cancer is poor. Clinical trial results to date suggest that this patient population could benefit from selective RET inhibition. Nevertheless, advanced/metastatic thyroid cancer can be classified as a rare disease and optimal patient management is often difficult to put in place. Generally, in clinical practice there is a long period between diagnosis and an advanced stage of thyroid cancer – often decades – during which patients are seen by multiple specialists. Ensuring selective personalised treatment is challenging, and a multidisciplinary team approach is essential not to lose patients along the way.

The advent of specific RET inhibitors with improved tolerability will most likely change the timing for initiation of these agents, making them the first systemic therapy in patients with advanced/metastatic disease. In this context, selpercatinib was approved in September 2022 for use as first line in agnostic advanced *RET*-mutant MTC in the EU (76). In addition, more patients on a wait and watch strategy might be able to receive a specific inhibitor, although it is suggested that eligibility criteria should be strictly defined. The presence of disease progression and symptoms, and increased biomarkers such as calcitonin, might help physicians in their treatment decision.

## 8 Future directions

The first head-to-head trials of RET-selective inhibitors and standard of care in treatment-naïve settings are planned or underway. The ongoing trial, LIBRETTO-531 (NCT04211337), is investigating the safety and efficacy of selpercatinib compared with standard treatment (cabozantinib or vandetanib) in MKI-naive patients with *RET*-mutant MTC that cannot be resected or has metastasised (90). Patients who are assigned to the standard treatment and discontinue due to either progressive disease or intolerability have the option to potentially crossover to selpercatinib when a progression according to radiological criteria (RECIST) is observed (91). The results of this trial will inform the sequence of initiation of this novel selective RET inhibitor, and pending are trials of selpercatinib and pralsetinib early in the course of thyroid cancer.

While it is expected that the timing of initiation of specific RET inhibitors will change towards earlier use as systemic therapy in advanced/metastatic settings, studies are also ongoing in neo-adjuvant settings. In this context, selpercatinib (NCT04759911) and the MKI lenvatinib (NCT04321954), initiated prior to surgery, are being investigated in clinical studies in patients with *RET*-altered thyroid cancer and will provide answers soon. Selpercatinib showed promise in the neoadjuvant setting in a patient with initially unresectable, metastatic, *RET*-mutated MTC who was treated on a single patient protocol (92). The 20-year-old patient achieved a 51.5% RECIST response on selpercatinib and was able to undergo complete surgical resection of the primary tumour. Selpercatinib treatment was resumed post-surgically and the patient remained locoregionally disease-free with stable metastatic disease (after an initial partial response) 21 months after starting therapy. The possibility to reinduce radioiodine uptake in DTC cases previously demonstrated to have lost such ability has been also explored by some authors (93–96). These results should be confirmed in a prospective clinical trial even if the low number of cases represents a big limit to the enrolment.

As seen with other targeted therapies, such as the MKIs (97), resistance to RET inhibition can emerge through potential escape from RET targeting (98, 99). Although recurrent *RET* kinase domain mutations play a role, most resistance appears to derive from RET-independent mechanisms (99). Investigations into the molecular determinants indicate that the key mechanisms of primary and acquired selective RET inhibitor resistance are mediated by MAPK pathway reactivation, and at the individual level this may be due to multiple distinct, yet partially overlapping mechanisms (97, 98). In one study, selpercatinib resistance was shown to be mediated by acquired RET G180 solvent front mutations (100). Hence, sequential RET-directed therapy may require combination treatment with inhibitors targeting alternative MAPK effectors (98). Evaluations of the molecular and pathological patterns of treatment resistance following RET selective inhibitor therapy in patients with MTC have shown (i) acquisition of resistance mutation is the primary cause of progressive disease, which can be captured by liquid biopsy, and (ii) bypass mutations of resistance may occur at a substantially higher frequency (80%) than on-target resistance mutations (101). Further developments regarding resistance are awaited with interest.

Second-generation RET inhibitors with different properties are in development, which may be of particular interest for the management of patients with progression despite anti-RET therapy. TPX-0046, a next generation RET inhibitor that is structurally differentiated and potent against a broad range of mutations, has entered a first-in-human phase I/II study (ClinicalTrials.gov Identifier: NCT04161391) after IND-enabling pre-clinical studies. Other selective RET inhibitors in development include BOS172738 (NCT03780517), LOXO-260 (NCT05241834) and TAS0953/HM06 (in pre-clinical development). Early nonclinical studies with BiDAC™ (bifunctional degradation activating compounds) a RET inhibitor from C4 therapeutics, and second-generation selective RET inhibitors from KinaRx and LOXO Oncology (LOX-18228 and LOX-19260) are also ongoing.

Nevertheless, combination therapy might prove to be the most effective strategy for patients with *RET*-driven advanced thyroid cancer. Multimodal/combination therapy trials combining several agents with synergistic approaches may provide therapies with more potent clinical utility, such as concurrent inhibition of RET and signalling pathways, or the more general mechanism of tumour progression (13).

## 9 Conclusions

The molecular profiling of thyroid cancers and development of targeted therapies have revolutionised the therapeutic landscape for patients with advanced thyroid cancer. Additionally, updated guidelines for the genomic profiling of patients with advanced and progressive thyroid cancer will facilitate molecular-targeted treatment according to driver gene alterations, such as *RET* mutation or fusion. Prior to the development of MKIs such as sorafenib, lenvatinib and cabozantinib, there was no effective treatment for advanced RAI-refractory DTC or *RET*-altered MTC. MKIs targeting RET in addition to multiple other kinases have shown favourable activity in these thyroid cancer subtypes; however, the potency of MKI RET inhibition is limited by off-target toxicity, predominantly due to targeting of the non-RET kinases, and high rates of dose reduction and drug discontinuation. The selective RET inhibitors selpercatinib and pralsetinib are an advance in the treatment of *RET*-driven thyroid cancer. These RET-selective inhibitors have demonstrated potent efficacy and favourable toxicity profiles in clinical trials and are now a new therapeutic option for patients with *RET*-altered thyroid cancers in the clinical setting. These advances lead us to recommend that genetic testing to detect a *RET* alteration should be offered before initiation of systemic therapy; with the approval of selpercatinib, RET inhibitors should be considered as first-line therapy for MTC. Finally, the importance of a multidisciplinary team approach must be emphasised as it is essential for the optimal management of patients with advanced and/or metastatic thyroid cancer.

## Author contributions

RE and MK were involved in the conception of the work; TP and NF were involved in the conception and design of the work. All authors contributed to the article and approved the submitted version. 
